# Multi-compartment head modeling in EEG: Unstructured boundary-fitted tetra meshing with subcortical structures

**DOI:** 10.1371/journal.pone.0290715

**Published:** 2023-09-20

**Authors:** Fernando Galaz Prieto, Joonas Lahtinen, Maryam Samavaki, Sampsa Pursiainen

**Affiliations:** Computing Sciences, Faculty of Information Technology and Communication Sciences, Tampere University, Tampere, Pirkanmaa, Finland; Universita Politecnica delle Marche Facolta di Ingegneria, ITALY

## Abstract

This paper introduces an automated approach for generating a finite element (FE) discretization of a multi-compartment human head model for electroencephalographic (EEG) source localization. We aim to provide an adaptable FE mesh generation tool for EEG studies. Our technique relies on recursive solid angle labeling of a surface segmentation coupled with smoothing, refinement, inflation, and optimization procedures to enhance the mesh quality. In this study, we performed numerical meshing experiments with the three-layer Ary sphere and a magnetic resonance imaging (MRI)-based multi-compartment head segmentation which incorporates a comprehensive set of subcortical brain structures. These experiments are motivated, on one hand, by the sensitivity of non-invasive subcortical source localization to modeling errors and, on the other hand, by the present lack of open EEG software pipelines to discretize all these structures. Our approach was found to successfully produce an unstructured and boundary-fitted tetrahedral mesh with a sub-one-millimeter fitting error, providing the desired accuracy for the three-dimensional anatomical details, EEG lead field matrix, and source localization. The mesh generator applied in this study has been implemented in the open MATLAB-based Zeffiro Interface toolbox for forward and inverse processing in EEG and it allows for graphics processing unit acceleration.

## Introduction

Non-invasive electroencephalography (EEG) source localization [1] is the process of identifying active regions of the brain through a set of measurements. This is accomplished by solving an inverse problem [2], where the measured EEG signals constitute the data and the distribution of electric brain activity the unknown. The process involves modeling the head as a volume conductor and solving the forward problem [3–5], which refers to the calculation of the electric potential distribution that would be generated by a known distribution of sources within the brain. The inverse problem can be solved using various techniques to estimate the distribution of sources that best explain the measured EEG signals on the scalp.

As a forward modeling technique, we focus on the finite element method (FEM) [6] which, in medical sciences, is advantageous for modeling the human head along with its electromagnetic fields since it allows both surface- and volume-based mesh fitting. However, the internal tissue layers of the human head, which can be described by a set of surface grids similarly as computer-assisted design (CAD) [7], poses a challenging task for volumetric FE mesh generation due to their complex geometrical properties.

While well-known heuristic FE mesh generators, such as TetGen [8], Netgen [9], and Gmsh [10], primarily aim at reconstructing non-intersecting surfaces, they are not deemed applicable for reconstructing complex brain geometry with many thin and strongly folded layers in their standard form unless a customized set of user-defined properties is supplied to the mesh generation routine. MATLAB-based iso2mesh toolbox represents an example of such a customized approach; as it utilizes TetGen and has been demonstrated to generate a high-quality tetrahedral mesh for a given five-compartment MRI-based segmentation composed of the scalp, skull, cerebrospinal fluid (CSF), grey matter, and white matter, [11], which are often distinguished by the FE models applied in non-invasive brain source imaging, see, e.g., [12]. More compartments can be added, e.g., through unfitted meshing, which has been exhibited in the recent studies introducing the open Zeffiro Interface (ZI) [13] and Duneuro [14] toolboxes. However, the quest of fitting FE mesh to an arbitrary segmentation remains open.

The meshing routine introduced in this study has been designed for an adaptable number of compartments to satisfy the requirements of applied EEG studies. We aim to generate an unstructured boundary-fitted FE mesh with a comprehensive set of subcortical brain structures in addition to the aforementioned five tissue types. The recent findings of [15–18] highlight the importance of modeling subcortical structures, where the brain activity is known to be only weakly distinguishable based on non-invasive data and exceptionally sensitive to noise and modeling inaccuracies. Of these studies, subcortical FE meshing has been considered in [17], which utilized an unfitted approach to discretize a full set of compartments within the segmentation, and in [18], which was limited to three- to six-compartment mesh approximations generated via iso2mesh.

Our code implementation has been integrated as part of the MATLAB-based forward and inverse processing tool ZI which we apply to investigate the effect of meshing accuracy on both simulated and experimental non-invasive EEG source imaging. In particular, we measure the accuracy of the EEG lead field matrices obtained with unstructured boundary-fitted vs. unfitted meshes and perform a series of EEG source localization experiments to assess the quality of those matrices. As a reference, we used a three-layered spherical Ary model [19], and an eighteen-compartment MRI-based head model [20] segmented via FreeSurfer software suite [21]. For source localization [22], we use the minimum norm estimate (MNE) [23], the standardized low-resolution brain electromagnetic tomography (sLORETA) [24], and the dipole scan method, also known as the deviation scan [25].

We explore the 14.0 and 22.0 millisecond median nerve stimulus responses reconstructed from experimental Somatosensory Evoked Potential (SEP) data [17] using a combination of sLORETA and Gaussian mixture modeling (GMM) [26]. We also examine the computational performance of the code, especially, graphics processing unit (GPU) acceleration which is utilized to speed up mesh labeling and surface extraction processes. The results demonstrate that our approach could achieve a sub-one-millimeter fitting error, which was found to provide the desired accuracy for the three-dimensional anatomical details, EEG lead field matrix, and source localization.

## Materials and methods

### EEG forward model

In EEG, an unknown primary source current distribution ${\vec{J}}_{p}$ is to be localized given a vector **y** containing point measurements of the electric potential distribution *u*. Given an electric potential ${\vec{J}}_{p}$, solution to the second-order partial differential equation is obtained as

$$\begin{array}{c} \nabla \cdot \left(\sigma \nabla u\right)=\nabla \cdot {\vec{J}}_{p},\;\text{in}\;\Omega \;\text{and}\;\left(\sigma \nabla u\right)\cdot \vec{n}=0\;\text{on}\;\partial \Omega , \end{array}$$ (1)

where Ω denotes a head model, ∂Ω is its boundary with outward normal vector $\vec{n}$, and *σ* is the electric conductivity distribution interfering with *u*. Integrating (1 by parts, yields the weak form $\int_{\Omega }\sigma \nabla u\cdot \nabla v\;\text{d}\Omega =\int_{\Omega }\left(\nabla \cdot {\vec{J}}_{p}\right)v\;\text{d}\Omega$ which is required for all Lebesque square-integrable test functions *v* ∈ *H*^1^(Ω) = {*v* ∈ *L*_2_(Ω): ∂_*i*_*v* ∈ *L*_2_(Ω)} with first-order weak partial derivatives ∂_*i*_*v* ∈ *L*_2_(Ω), i.e., the functions of Sobolev space *H*^1^(Ω) [4]. Approximating *u* and ${\vec{J}}_{p}$ with finite sums $u^{\left(h\right)}=\sum_{i=1}^{n}\;z_{i}\psi_{i}$, *ψ*_*i*_ ∈ *H*^1^(Ω), and ${\vec{J}}_{p}^{\left(h\right)}=\sum_{j=1}^{m}x_{j}{\vec{w}}_{j}$, the weak form of (1) can be expressed in a Ritz-Galerkin discretized form

$$\begin{array}{c} \text{Az}=\text{Gx}, \end{array}$$ (2)

where **z** = (*z*_1_, *z*_2_, …, *z*_*n*_) and **x** = (*x*_1_, *x*_2_, …, *x*_*m*_) represent the coefficient vectors of the FE-discretized electric potential and primary current distribution, and the matrices A and G discretize the partial differential operators on the left and right-hand side, respectively [4]. Solving (2) with respect to z and taking into account that **y** can be obtained by multiplying **z** with a restriction operator **R**, i.e., **y** = **Rz**, implies the linear forward model

$$\begin{array}{c} \text{Lx}=y, \end{array}$$ (3)

where **L** = **RA**^−1^**G** is the lead field matrix. To obtain such a matrix, we apply the divergence conforming H(div) source model in which ${\vec{J}}_{p}$ is assumed to belong to the space $H\left(\text{div};\Omega \right)=\{{\left(\vec{w}\right)}_{i}\in L_{2}\left(\Omega \right),\text{for}\;i=1,2,3\;:\;\nabla \cdot \vec{w}\in L_{2}\left(\Omega \right)\}$ [27] of vector fields with *L*_2_(Ω)-integrable components and divergence. Divergence conforming sources ${\vec{w}}_{j}\in H\left(\text{div};\Omega \right)$ are dipole-like; each source is curl-free and characterized by its location, orientation, and strength [4].

#### Inverse problem

The Eq (3) poses an ill-posed inverse problem with respect to the unknown activity **x**, and the source currents corresponding to the entries of **x** are assumed to be dipolar [1]. Since the non-unique solution of this problem is subject to the applied inverse approach, we reconstruct **x** use three different techniques, MNE, sLORETA, and the dipole scan method, which are briefly reviewed below.

#### Minimum norm estimate

MNE [23] is evaluated as the solution to the following regularized minimization problem:

$$\begin{array}{c} \underset{x}{\text{min}}\{{\left\|C^{-1/2}(Lx-y)\right\|}^{2}+{\left\|\Sigma^{-1/2}x\right\|}^{2}\}, \end{array}$$ (4)

where **C** and **Σ** denote the noise and prior covariance matrix. This problem (4) can be associated with a Bayesian setting in which both the prior and likelihood have Gaussian densities. We select prior and noise covariance according to [17] in which the SEP dataset of this study was investigated. That is, **C** and **Σ**, are assumed to be proportional to identity, i.e., **C** = *ν* **I** and **Σ** = *θ*_0_**I**; the noise standard deviation *ν* is assumed to be 3% (-30 dB) of the maximum data amplitude max_*i*_ |*y*_*i*_|; and the prior standard deviation *θ*_0_ is chosen to be 20 dB with respect to the noise level when measured as in [28]. Consequently, the minimization problem (4) obtains the classical Tikhonov-regularized form min_**x**_{‖**Lx** − **y**‖^2^ + λ^2^ ‖**x**‖^2^} with the regularization parameter λ = *θ*_0_/*ν*. The noise assumption above applies throughout this study including the creation of the synthetic data.

#### Standardized low-resolution brain electromagnetic tomography

sLORETA [24] can be interpreted as re-scaled MNE; The solution of the MNE minimization problem is found first after which it is standardized by scaling. Assuming that the minimization problem is of the above Tikhonov-regularized form, the scaling corresponds to dividing each entry of MNE by its respective diagonal entry in **S** = **L**^*T*^(**LL**^*T*^ + λ^2^**I**)^−1^**L**. An sLORETA reconstruction can be expected to have an enhanced depth balance compared to MNE, as the energy of the standardized reconstruction is not affected by the variable amplitude of the lead field in different parts of the domain, where the source reconstruction is found.

#### Dipole scan

The dipole scan method [25] evaluates the subsequent goodness-of-fit (GOF) *g* between the data and a dipole source at the *ℓ*-th position of the space as

$$\begin{array}{c} g=1-\frac{\|y-L_{\ell }L_{\ell }^{†}{y\|}^{2}}{{\left\|y\right\|}^{2}}, \end{array}$$ (5)

where **L**_*ℓ*_ is the submatrix of **L** containing the associated source(s) at the *ℓ*-th position. The reconstruction is a GOF distribution covering the whole source space.

### Tetra meshing

**Fig 1:**
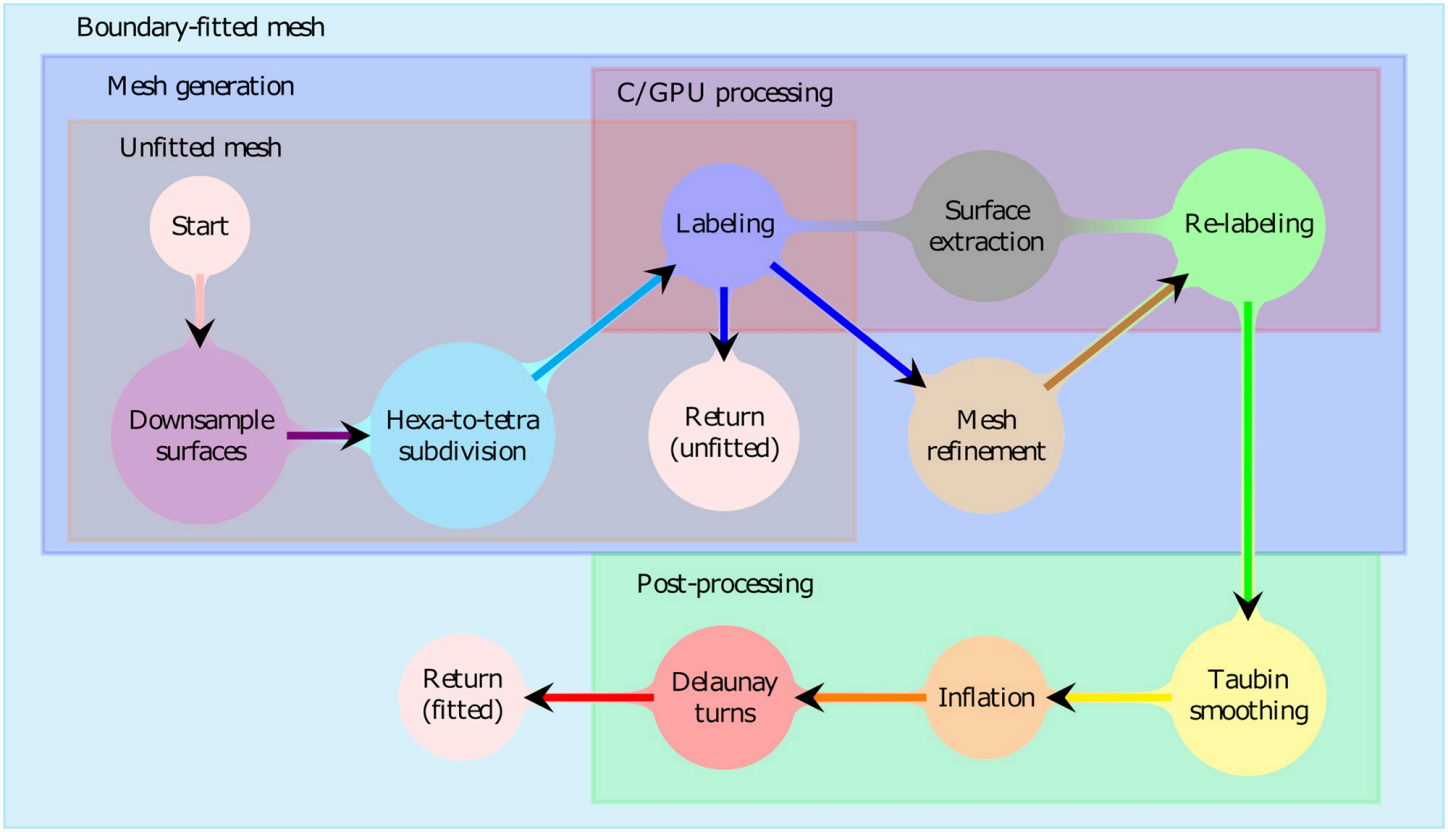
Mind map of the meshing process. The unfitted mesh is obtained after the first labeling stage, while the boundary-fitting process includes additional processing stages for refinement, re-labeling, smoothing, inflation, and optimization via Delaunay turns. A graphics processing unit (GPU) can be applied to accelerate the solid angle labeling and re-labeling stages as well as the surface extraction stage which finds the compartment boundaries after labeling. The re-labeling process is run recursively as long as one or more compartment labels change their value. A pseudocode of this mind map is provided in data in S1 Appendix.

Our approach to achieving precise FE meshing with subcortical brain components involves two stages. The first one, *mesh generation*, comprises creating an initial tetrahedral mesh by subdividing a regular hexahedral point lattice, mesh refinement, as well as recursive solid angle labeling and re-labeling steps performed with respect to a given multi-compartment surface segmentation as detailed in [29]. The second stage, *post-processing*, involves surface smoothing, inflation, and optimization tasks. A mind map conceptualizing the meshing algorithm is depicted in Fig 1 and as a pseudocode in data in S1 Appendix.

#### Surface downsampling

In the FE mesh generation process, redundant computations during the labeling phase are avoided by first evaluating the point density of the surface grids, e.g. STL files, that determine the head segmentation. If the calculated density exceeds the given FE mesh resolution, the segmentation is downsampled to match the resolution of the FE mesh, which will reduce the computational cost of labeling.

#### Unfitted mesh

**Fig 2:**
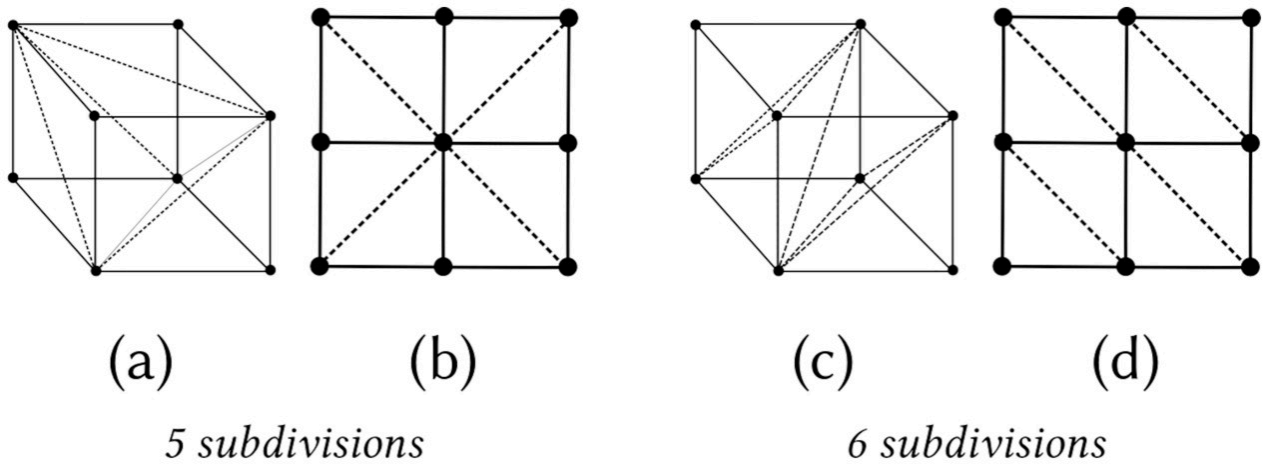
Hexahedral-to-tetrahedral subdivision. Schematic illustration of a (a) hexahedron subdivided into five tetrahedra [30]. We chose this subdivision strategy as the resulting (planar) alternating pattern of faces and edges in adjacent hexahedra (b) is advantageous to reduce lattice-dependent deficiencies in the mesh. In contrast, a subdivision into six tetrahedra (c) results in a non-alternating pattern creating potential geometrical bias, e.g., a directional bias of smoothing (d).

The meshing process initiates by setting a regular hexahedral lattice in the domain. Each hexahedron is subdivided into five tetrahedral elements (Fig 2), i.e., the lowest subdivisions possible [30]. This subdivision strategy provides several benefits: it leads towards an alternating direction of the diagonal sub-dividing the edges over the adjacent hexahedra, restricts the generation of weighted directional bias affecting further mesh manipulation operations (e.g., smoothing, inflation, optimization), and minimizes the total amount of computer memory required to support the dataset.

#### Mesh refinement

**Fig 3:**
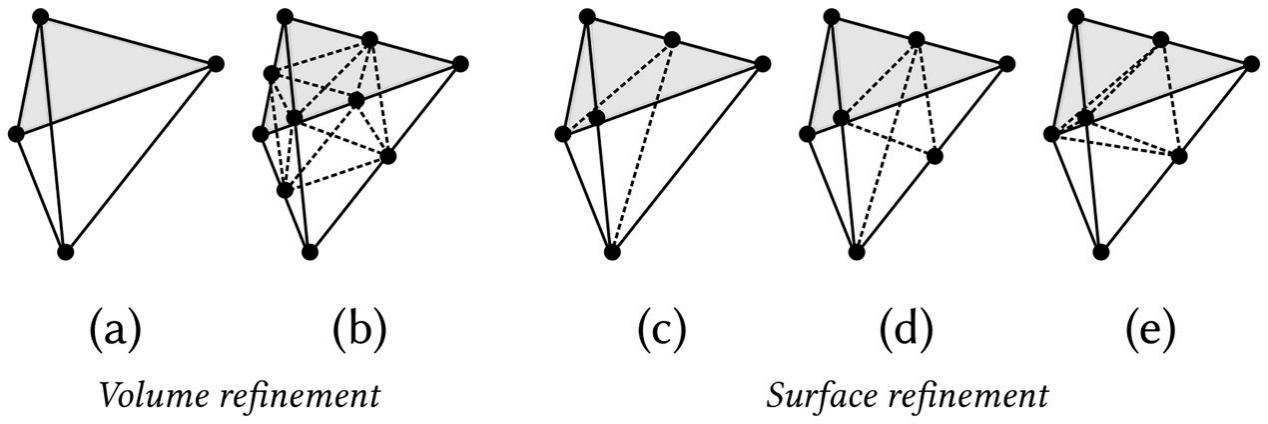
Tetrahedra refinement. Schematic illustration of the volume and surface refinement. In volume refinement, each tetrahedral element (a) in the given subdomain *V* of Ω is subdivided into eight tetrahedra (b). The refined volume *V* is connected to the remaining part of the domain Ω\*V* via surface refinement where the elements can have one to three edges subdivided on the face adjacent to the boundary ∂*V*, leading to tetrahedral subdivisions (c, d, e), respectively. A refinement with respect to a boundary ∂*V* can be defined as two similar refinements on both sides of ∂*V*.

The mesh can be refined to a given subdomain *V* or its boundary ∂*V* (Fig 3). The elements in *V* are subdivided into eight triangular faces. In contrast, in Ω\*V*, tetrahedra having nodes adjacent to the boundary are subdivided according to edges shared with ∂*V*.

#### Labeling

**Fig 4:**
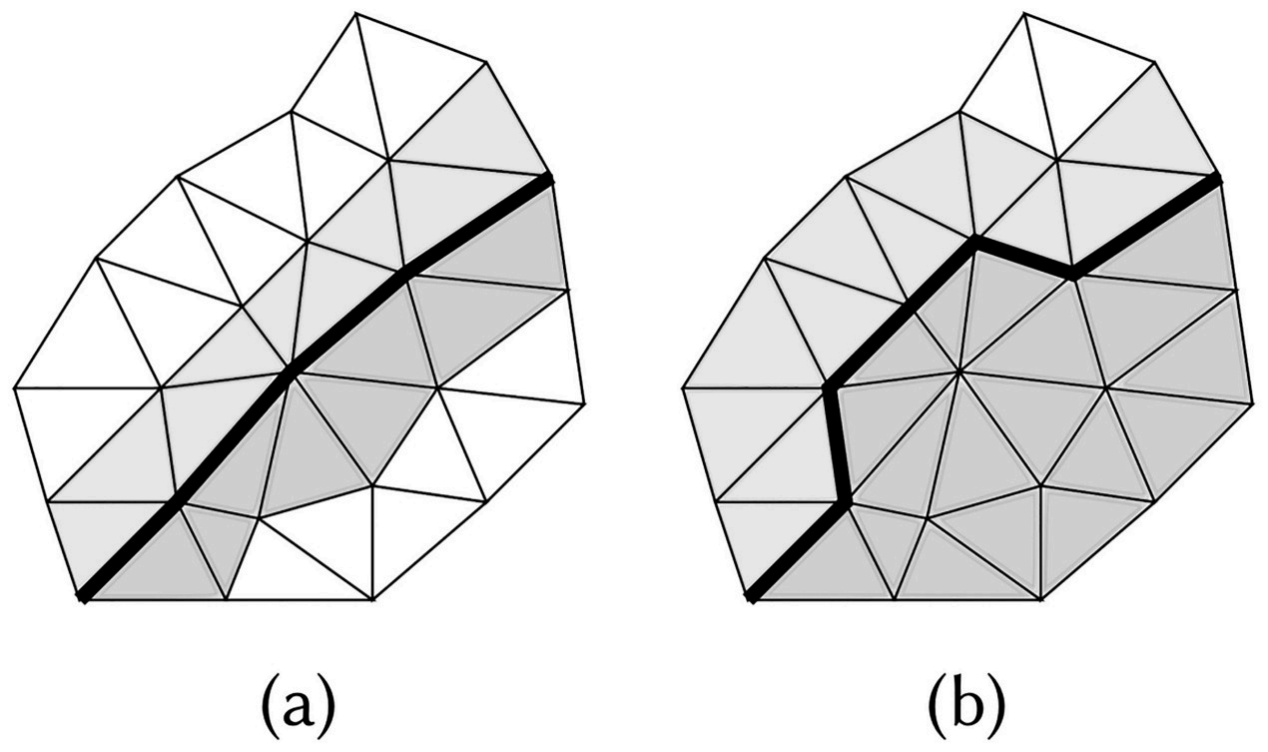
Recursive labeling condition. Recursive solid angle labeling process re-labels the tetrahedra (triangles) adjacent to a given boundary ∂*V* (black curve) between two different FE mesh compartments (light and dark grey). The re-labeling process is performed recursively until ∂*V* does not change between two consecutive recursion steps, as it does between (a) and (b).

To obtain the compartment of a given element, we apply the solid angle labeling method [29], i.e., a mesh node at position ${\vec{r}}_{i}$ associated with the integral

$$\begin{array}{c} s_{i}=\frac{1}{4\pi }\int_{\partial S}\left(\vec{r}-{\vec{r}}_{i}\right)\cdot \vec{n}\;\text{d}A, \end{array}$$ (6)

where ∂S is a closed segmentation boundary, d*A* is a surface area differential, and $\vec{n}$ is the normal vector at the point $\vec{r}$. The integral *s*_*i*_ defines the ratio between the solid angle and the angle covered by ∂S with respect to ${\vec{r}}_{i}$; a point is enclosed by ∂S if *s*_*i*_ >= *T* with *T* denoting a given threshold value between 0 and 1. Tetrahedra with four nodes inside ∂S are labeled as the elements contained within the surface *S*. The labeling and re-labeling processes are performed as follows:

1. Labeling: All nodes within a tetrahedral mesh are labeled using the solid angle method, covering all segmentation boundaries ∂S, i.e., the (fixed) surface grids of the given head segmentation.
2. Re-labeling: Re-labeling sharpens each compartment boundary ∂*V* in the tetrahedral mesh after a mesh refinement. The solid angle integral *s* is re-evaluated for the nodes shared by the tetrahedra adjacent to ∂*V*. The labels of those tetrahedra are updated, potentially changing ∂*V*, which approximates the corresponding (fixed) segmentation boundary ∂S. The process is repeated recursively until achieving convergence, i.e., when the status of one or more nodes remains unchanged (Fig 4).

Labeling proceeds from the innermost compartment to the outermost one according to a user-defined *a priori* ordering. Whenever an intersecting segmentation boundary occurs, the one that comes first in the layer hierarchy will take priority. A temporal bounding box, enclosing the head model acts as the outermost compartment to prevent any adverse shrinkage effects that might take place on the external surface during the different mesh processing steps. Once the unfitted mesh has been generated, labeled, refined, and re-labeled, the resulting mesh is post-processed via smoothing, inflation, and optimization to enhance the fit between any FE mesh-based compartment surface ∂*V* and its associated (fixed) segmentation boundary ∂S.

### Post-processing

The mesh generation stage has been designed to conform to a head segmentation with closed boundaries. It enables the targeted tissue structures to (I) be complex-shaped, such as the cerebral cortex and subcortical nuclei, (II) have thin layers, such as the scalp, and/or (III) pose significant electric conductivity contrasts, such as the skull [31].

#### Smoothing

As a smoothing technique, we employ Taubin’s method [32], which performs alternating Laplacian forward and backward smoothing steps to smooth the mesh, and to reduce shrinkage, respectively. Both volume and tissue boundaries are smoothed using as

$$\begin{array}{ccc} {\vec{x}}_{i}^{\left(k+1/2\right)} & = & {\vec{x}}_{i}^{\left(k\right)}+\lambda \sum_{j\in N_{i}}x_{j}^{\left(k\right)}, \end{array}$$ (7)

$$\begin{array}{ccc} {\vec{x}}_{i}^{\left(k+1\right)} & = & {\vec{x}}_{i}^{\left(k\right)}-\mu \sum_{j\in N_{i}}x_{j}^{\left(k+1/2\right)}, \end{array}$$ (8)

where λ < 1 and *μ* < 1 are the smoothing parameters, and $N_{i}$ is the index set containing the *i*-th node together with its neighbors. In volumetric smoothing, all the neighbors connected by an edge with ${\vec{x}}_{i}^{\left(k\right)}$ are included in $N_{i}$. The stopping criterion for smoothing is set as

$$\begin{array}{c} \frac{\|{\vec{x}}^{\left(k+1/2\right)}-{\vec{x}}^{\left(k\right)}{\|}_{2}}{\|{\vec{x}}^{\left(0\right)}{\|}_{2}}\leq \xi , \end{array}$$ (9)

where *ξ* is a user-defined value.

#### Inflation

Following the labeling procedure, each FE mesh-based compartment surface ∂*V* becomes enclosed by its associated (fixed) segmentation boundary ∂S as each tetrahedron adjacent to ∂*V* now has four nodes inside ∂S. Once those four nodes have been enclosed, they are lifted through an inflation sequence. This method involves first identifying the edges intersecting with ∂S, then locating the intersection point, and finally moving the nodes of ∂*V* along their associated edges towards those points. The degree of inflation for a given intersecting edge is determined using the following formula:

$$\begin{array}{c} {\vec{x}}_{i}^{\left(1\right)}={\vec{x}}_{i}^{\left(0\right)}+\zeta d\vec{e}, \end{array}$$ (10)

where $\vec{e}$ is a unit vector parallel to the edge, *d* is the distance between ${\vec{x}}_{i}$ and ∂S, and *ζ* < 1 is a parameter that controls the level of the inflation.

#### Delaunay turns

Optimization via Delaunay turns is applied to guarantee that the generated elements have a sufficient condition *κ*, which for each tetrahedron is determined by the following ratio of its volume $V_{T}$ to the length of its longest edge $\ell_{T}^{\left(\text{max}\right)}$:

$$\begin{array}{c} \kappa =\frac{V_{T}}{\ell_{T}^{\left(\text{max}\right)}}. \end{array}$$ (11)

The optimization process looks for tetrahedra with inverted elements containing negative determinants. These elements are fixed by moving any node outside of the outer surface of its related supernode back inside the surface of the set that includes all the edge-sharing neighbor nodes. After fixing all negative determinants, the optimization performs Delaunay turns [33] for each pair of adjacent tetrahedra in which at least one tetrahedron has condition less than a specific threshold (*κ* < *τ*). The orientation of the shared face is determined by the tetrahedron with the smaller condition.

## Numerical modeling

### Spherical three-layer Ary model

The spherical three-layer Ary model [19] is composed of three concentric spheres with radii of 87, 92, and 100 mm (millimeter) and conductivities of 0.33, 0.0042, and 0.33 S/m (Siemens per meter), respectively. The steep contrast between the adjacent brain and skull layers, analyzed using a universal heuristic mesh generator, such as in [34], poses a challenge for forward simulation and dipole localization. A semi-analytical solution was obtained by finding the Berg parameters as described in [35]. To simulate scalp measurements, 180 measurement points were evenly spread over the external surface.

### MRI-based head model and segmentation

The MRI-based head model was obtained from an openly available dataset (https://doi.org/10.5281/zenodo.3888381) [20] including MRI data of a healthy right-handed 48 years old adult subject. The MRI data were segmented via FreeSurfer Software Suite’s (https://surfer.nmr.mgh.harvard.edu/) standard surface reconstruction procedure yielding 18 tissue compartments of which the subcortical ones were based on FreeSurfer’s Aseg Atlas. The tissue conductivities were set according to their literature reference values [31]: 0.33 S/m for the scalp, 0.0064 S/m for the skull, 1.79 S/m for CSF, 0.33 S/m for grey matter, and 0.14 S/m for white matter. As for the subcortical compartments, 0.33 S/m was chosen according to [17, 36].

### Meshing experiments

We examine tetra meshing using (i) a regular unfitted mesh obtained by subdividing a regular hexahedral 1.0 mm lattice into a tetrahedral one, and (ii) an unstructured boundary-fitted mesh corresponding to hexahedral 3.0, 2.0, and 1.3 mm lattices. The resolution of the boundary-fitted mesh was adjusted compartment-wise according to *a priori* knowledge of EEG forward modeling errors. A uniform refinement was performed on the boundaries of the compartments with neural activity, including the cerebrum, cerebellum, and subcortical active nuclei.

This is motivated, on one hand, by the relatively low physical 2.0–3.0 mm thickness of the adult human neocortex [37], which is close to the mesh resolution, and, on the other hand, by weak singularities of the electric potential *u*, occurring on the tissue boundaries, where the electric conductivity is discontinuous. A refined boundary layer can be, thereby, considered necessary, in particular, since a dipole-like source cannot be placed inside or next to a tetrahedron that touches such a boundary without significantly compromising the forward modeling accuracy [38].

In addition, we refined the boundaries of the skull and scalp compartments uniformly to avoid leakage effects [39] due to an overly coarse FE discretization compared to the thickness of these compartments, which is a few millimeters for healthy adults [19]. The smoothing parameters were set to λ = *μ* = 0.4, and the stopping criterion was set to *ξ* = 0.9 and *ξ* = 0.1 for volumetric and surface smoothing, respectively. The inflation parameter was chosen to be *ζ* = 0.05.

### Numerical analysis of FE mesh quality

The meshing accuracy is measured by evaluating the Euclidean distance between the surface of the tetrahedral approximation of the grey matter compartment and the original tissue boundary. We also measure the distribution of the element condition and evaluate three-dimensional mesh details visually. For the spherical model, we examine the lead field matrix accuracy for different eccentricities, i.e., relative distances from the origin in the brain compartment, using the the following relative difference (RDM) and magnitude (MAG) measures:

$$\begin{array}{ccc} \text{RDM}({\vec{J}}_{1},{\vec{J}}_{2}) & = & \;{\left\|\frac{{\vec{J}}_{1}}{{\left\|{\vec{J}}_{1}\right\|}_{2}}-\frac{{\vec{J}}_{2}}{{\left\|{\vec{J}}_{2}\right\|}_{2}}\right\|}_{2}, \end{array}$$ (12)

$$\begin{array}{ccc} \text{MAG}({\vec{J}}_{1},{\vec{J}}_{2}) & = & 1-\;(\frac{{\left\|{\vec{J}}_{1}\right\|}_{2}}{{\left\|{\vec{J}}_{2}\right\|}_{2}}), \end{array}$$ (13)

where ${\vec{J}}_{1}$ and ${\vec{J}}_{2}$ denote source approximations obtained via FEM-based and semi-analytical forward models, respectively. These models share the same set of source positions, while the set of orientations is Cartesian (see section EEG forward model) vs. random, respectively. The RDM can be interpreted as a topographical forward modeling error in terms of location and orientation, whereas the MAG concerns variations in amplitude.

### Source localization experiments

#### Source space

By employing the divergence conforming source model [4], we generated the EEG lead field matrix for 10,000 source positions which were distributed uniformly in the set of active compartments. The sources placed in the cerebrum were normally constrained parallel to the surface normal, following the normal orientation of the neurons and neural activity [40]. Otherwise, each source position in the lead field comprised three orientational (Cartesian) degrees of freedom.

#### Earth mover’s distance

To assess the accuracy of source localization estimates obtained for a given dipole, we employ a metric known as the Earth Mover’s Distance (EMD) [41]. This metric measures the minimum amount of work required to transform one mass distribution into another. The EMD was originally proposed as a distance function for comparing probability distributions in metric spaces [42, 43]. Essentially, the EMD calculates the amount of effort or energy needed to reshape one distribution into another. In our study, we use the EMD to evaluate the similarity between a reconstructed distribution and a dipole associated with a Dirac’s delta function (also known as a unit impulse). The EMD also allows us to calculate distributional source localization estimates for four sets of 18 dipoles placed at different eccentricities: 0.06, 0.29, 0.63, and 0.98. We compare the actual dipole source with the estimate to evaluate the similarity between them.

#### Gaussian mixture modeling

To estimate source localization in an MRI-based head model, we resort to clustering techniques since there is no reliable “ground truth” for this scenario. Specifically, we utilize Gaussian mixture modeling (GMM) [26] as the clustering method. The goal of GMM is to identify the primary concentration areas of the current density as a mixture or a superposition of Gaussian distributions. Our working hypothesis is that the estimate of ${\vec{J}}_{p}$ corresponds to either a unimodal or multimodal distribution of activity, with each distribution component’s mean location indicating the probable location of the corresponding neural activity. By assigning the weight

$$\begin{array}{c} w_{i}={\left\|\vec{J}\left({\vec{r}}_{i}\right)\right\|}_{2}^{2}{\left(\sum_{j=1}^{N}{\left\|\vec{J}\left({\vec{r}}_{j}\right)\right\|}_{2}^{2}\right)}^{-1}, \end{array}$$ (14)

with a given reconstruction $\vec{J}$ and with each source point ${\vec{r}}_{i}$, *i* = 1, 2, …, *N*, the GMM can detect a controlled number of neural activity clusters contained within $\vec{J}$. In order to determine the optimal number of clusters for the GMM technique, a deterministic procedure is used. This involves selecting the best fitting set for probability-thresholded Mahalanobis distance, which is a statistical measure that takes into account the covariance between variables and the mean values of different groups. This procedure recursively adds possible clusters to the configuration until the best fit is achieved. Once the possible clusters are determined, the GMM algorithm is applied to select the model with the lowest Bayes Information Criterion (BIC), which is a measure of the goodness of fit for a statistical model. Using both the Mahalanobis distance and the GMM algorithm with the BIC criterion, we can ensure that the estimated number of clusters is both statistically significant and provides the best representation of the data.

#### Somatosensory evoked potential data

We investigate a 74-channel non-invasive EEG dataset from a SEP experiment in which the median nerve of the right wrist was stimulated via monophasic square-wave electric pulses with 0.5-millisecond duration [20]. As suggested in [17], this dataset should allow distinguishing the originators of the early SEP responses in both subcortical and cortical regions. We estimate the originators of the P14/N14 and P22/N22 peak (occurring at 14 and 22 milliseconds post-stimulus, respectively) using sLORETA/GMM reconstructions and lead field matrices obtained with different FE meshes. The first one of these peaks originates in the medial lemniscus of the brain stem above the cuneate nucleus [44–48], while the second one has two origins: a cortical originator in the crown of either the pre- or post-central gyrus [25, 49, 50], and a subcortical one in the thalamus [51].

### Computing platform

In this study, the hardware applied consists of a Dell Precision 5820 Workstation with 256 GB RAM, a 10-core Intel i9–10900X CPU, and an NVidia Quadro RTX 4000 GPU. The software used for this study included MATLAB and ZI [13], which is an open-source software toolbox designed for effective FEM-based forward and inverse computations in MATLAB. The effectiveness of ZI partly comes from graphic processing unit (GPU) acceleration which has been enabled to reduce the computational effort of discretizing the complex geometry of the brain. With the incorporation of MATLAB’s Parallel Computing Toolbox, users of ZI can decompose the mesh generation framework and post-processing characteristics through (I) CPU parallelization, which involves running serial execution threads simultaneously, or (2) GPU parallelization, which decomposes the process into individually handled blocks of vectorized operations.

## Results

The downsampled surface segmentation (triangular) and volumetric (tetrahedral) FE mesh parameters for each model are presented in Table 1. The computing times (measured in seconds) for each case have been included in Tables 2 and 3. The finest FE mesh obtained with a 1.3 mm initial lattice resolution comprises 5.0 M (million) nodes and 27 M tetrahedrons for the sphere, and 7.5 M nodes and 40 M tetrahedrons for the MRI-based head model.

**Table 1:** The number of points and triangles in the (downsampled) surface grids vs. the number of nodes and tetrahedra in the finite element mesh for Ary sphere and the MRI-based head model.

	**Boundary-fitted**			**Unfitted**
**Property**	**3.0mm**	**2.0mm**	**1.3mm**	**1.0mm**
Points	34,773	42,284	42,284	42,284
Triangles	72,556	88,636	88,636	88,636
Nodes	607,184	1,833,945	5,003,931	4,269,175
Tetras	3,245,733	9,903,208	26,801,840	20,871,950
Size (MB)	707	1,860	5,092	6,612
(a) Ary sphere.				
	**Boundary-fitted**			**Unfitted**
**Property**	**3.0mm**	**2.0mm**	**1.3mm**	**1.0mm**
Points	46,555	104,684	186,057	186,057
Triangles	93,016	209,314	372,088	372,088
Nodes	837,029	2,517,262	7,526,427	4,798,880
Tetras	4,540,361	13,699,905	40,895,474	23,460,830
Size (MB)	1,297	2,812	7,254	6,463
(b) MRI-based mesh.				

**Table 2:** Total computing time (seconds) of (a) CPU- and (b) GPU-parallelized finite element meshing for Ary sphere (including post-processing methods, and MATLAB’s data handling).

	**Fitted**			**Unfitted**
**Function**	**3.0mm**	**2.0mm**	**1.3mm**	**1.0mm**
Surface extraction	32	114	335	249
Labeling	95	501	4810	7450
Refinement	7	23	64	0
Smoothing	62	50	151	0
Inflation	23	56	162	0
Optimization	15	223	801	0
Data handling	64	197	579	690
**Total**	298	1164	6902	8389
(a) CPU.				
	**Fitted**			**Unfitted**
**Function**	**3.0mm**	**2.0mm**	**1.3mm**	**1.0mm**
Surface extraction	32	116	354	247
Labeling	17	66	424	597
Refinement	8	24	65	0
Smoothing	15	51	153	0
Inflation	23	59	162	0
Optimization	62	243	777	0
Data handling	64	195	581	690
**Total**	221	754	2516	1534
(b) GPU parallelization.				

**Table 3:** Total computing time (seconds) of (a) CPU- and (b) GPU-parallelized finite element meshing for MRI-based model (including post-processing methods, and MATLAB’s data handling).

	**Fitted**			**Unfitted**
**Function**	**3.0mm**	**2.0mm**	**1.3mm**	**1.0mm**
Surface extraction	303	1124	2640	1493
Labeling	546	1528	11585	23947
Refinement	11	39	195	0
Smoothing	23	75	261	0
Inflation	90	247	714	0
Optimization	147	528	1925	0
Data handling	70	222	1711	642
**Total**	1190	3763	19031	26082
	**Fitted**			**Unfitted**
**Function**	**3.0mm**	**2.0mm**	**1.3mm**	**1.0mm**
Surface extraction	205	767	2416	1158
Labeling	184	451	1732	1706
Refinement	10	44	273	0
Smoothing	14	44	161	0
Inflation	78	202	553	0
Optimization	223	668	2084	0
Data handling	77	228	745	636
**Total**	791	2404	7946	3500

Of all mesh processing steps, most computing effort is spent on labeling, which can be accelerated using GPU parallelization; GPU labeling took, in most cases, less than 1/10 of the time consumed by the CPU. The labeling effect was most influential in the case of the unfitted mesh, where neither refinement nor post-processing was applied. In boundary-fitted meshing, the GPU accelerated total meshing time was close to 1/3–1/2 compared to the case of no acceleration. The post-processing phase (smoothing, inflation, and optimization) took roughly 30–40% of the total time required by the GPU-accelerated mesh generation.

**Fig 5:**
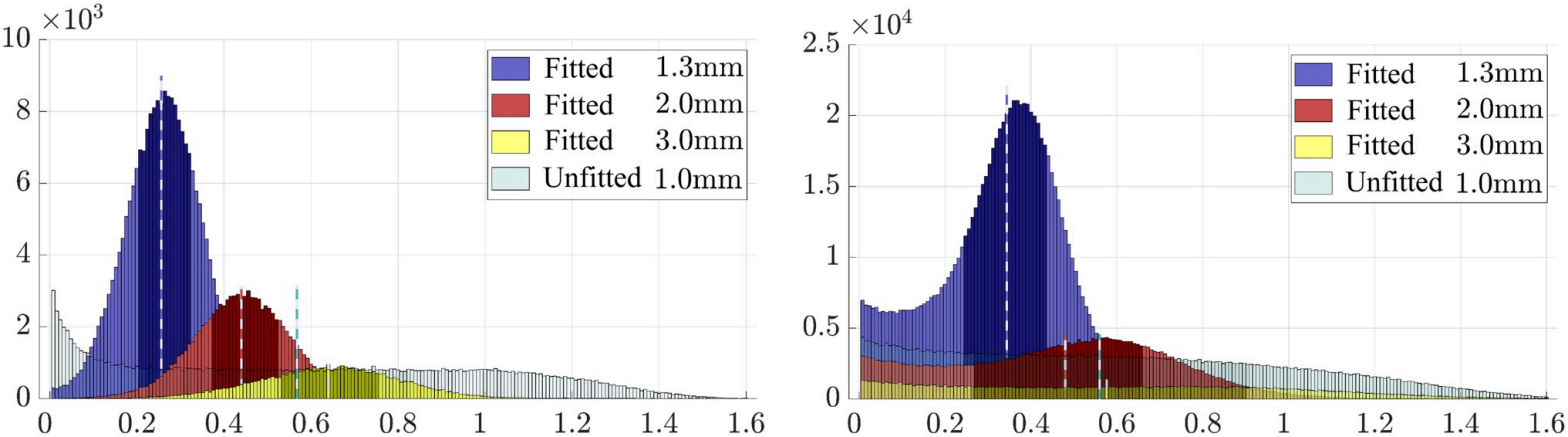
Fitting accuracy for the Ary sphere (A) and MRI-based head model (B) depicting the distance (millimeters) between the grey matter boundary in the FE mesh and in the segmentation.

The distance between the given original and FE meshing-based grey matter boundary is shown in Fig 5a and 5b. In the spherical case, the median of this distance is close to one-fourth of the initial mesh resolution, i.e., one-half of the refined mesh size in the vicinity of the boundary. For the MRI-based mesh, the errors were slightly greater and more dispersed, the median and spread (i.e. the inter-quartile range between the 25 and 75% quantiles) being smaller than the refined mesh size. The unfitted mesh corresponds to a comparably large spread following the absence of the refinement and post-processing steps.

**Fig 6:**
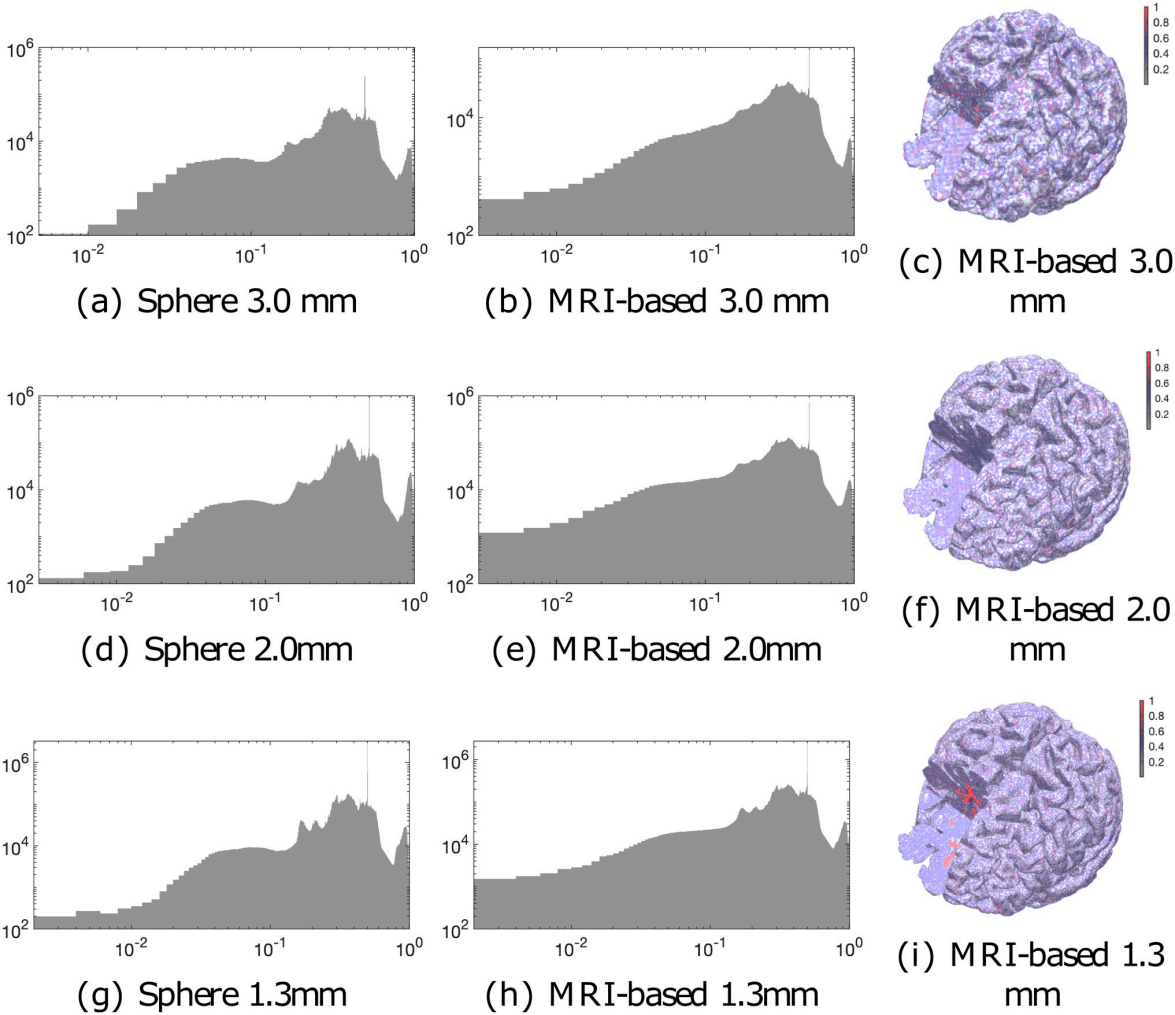
Element condition distribution. Element condition distributions of the boundary-fitted 3.0, 2.0, and 1.3 mm (millimeter) FE mesh are shown as a histogram for both the spherical Ary model (a, d, g) and the MRI-based head model (b, e, h). The spatial condition number mappings (c, f, i) for the MRI-based model show as expected that the non-refined internal parts of the mesh, in particular, the interior of the white matter, correspond to an elevated condition compared to the thin refined layers closer to the surface.

The element condition distributions are shown in Fig 6. While the distributions for the spherical and MRI-based meshes share similarities, MRI-based meshes have a greater proportion of the elements with conditions below 0.01, which we assume to be due to a greater number of smoothed surfaces. The spatial mappings in Fig 6 reveal that the non-refined internal parts, particularly the white matter interior, obtain an elevated condition compared to thin, refined layers closer to the surface.

**Fig 7:**
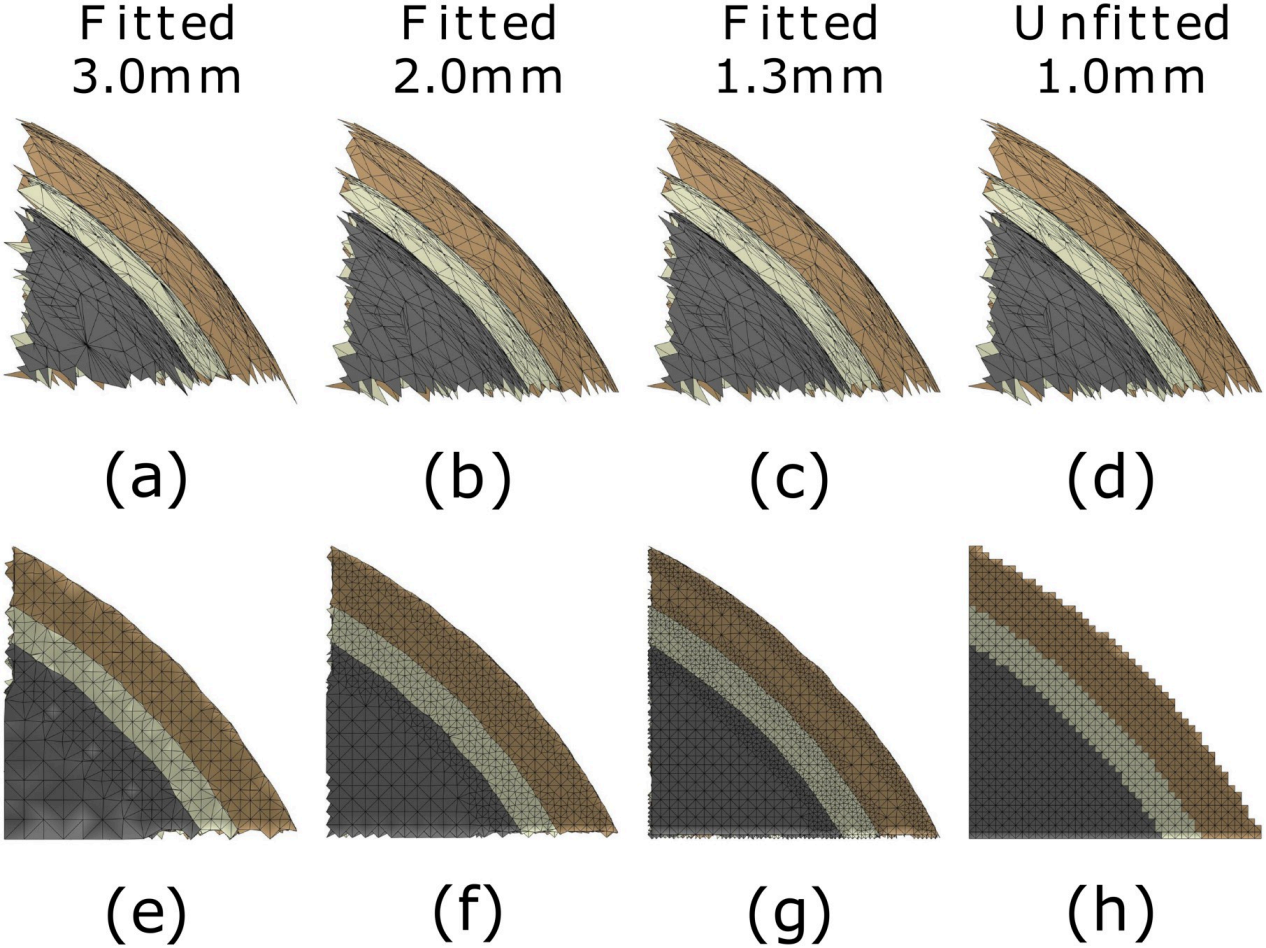
Mesh visualization: Ary sphere. Quadrants of the downsampled surface grids (a-c) and the boundary-fitted tetrahedral mesh (e-g) for 3.0, 2.0 and 1.3 mm (millimeter) mesh sizes, respectively. Surface grids (d) and unfitted tetrahedral mesh (h) for 1.0 mm mesh size are included for comparison. The presented layers are (top-bot): scalp (brown), skull (white), and grey matter.

**Fig 8:**
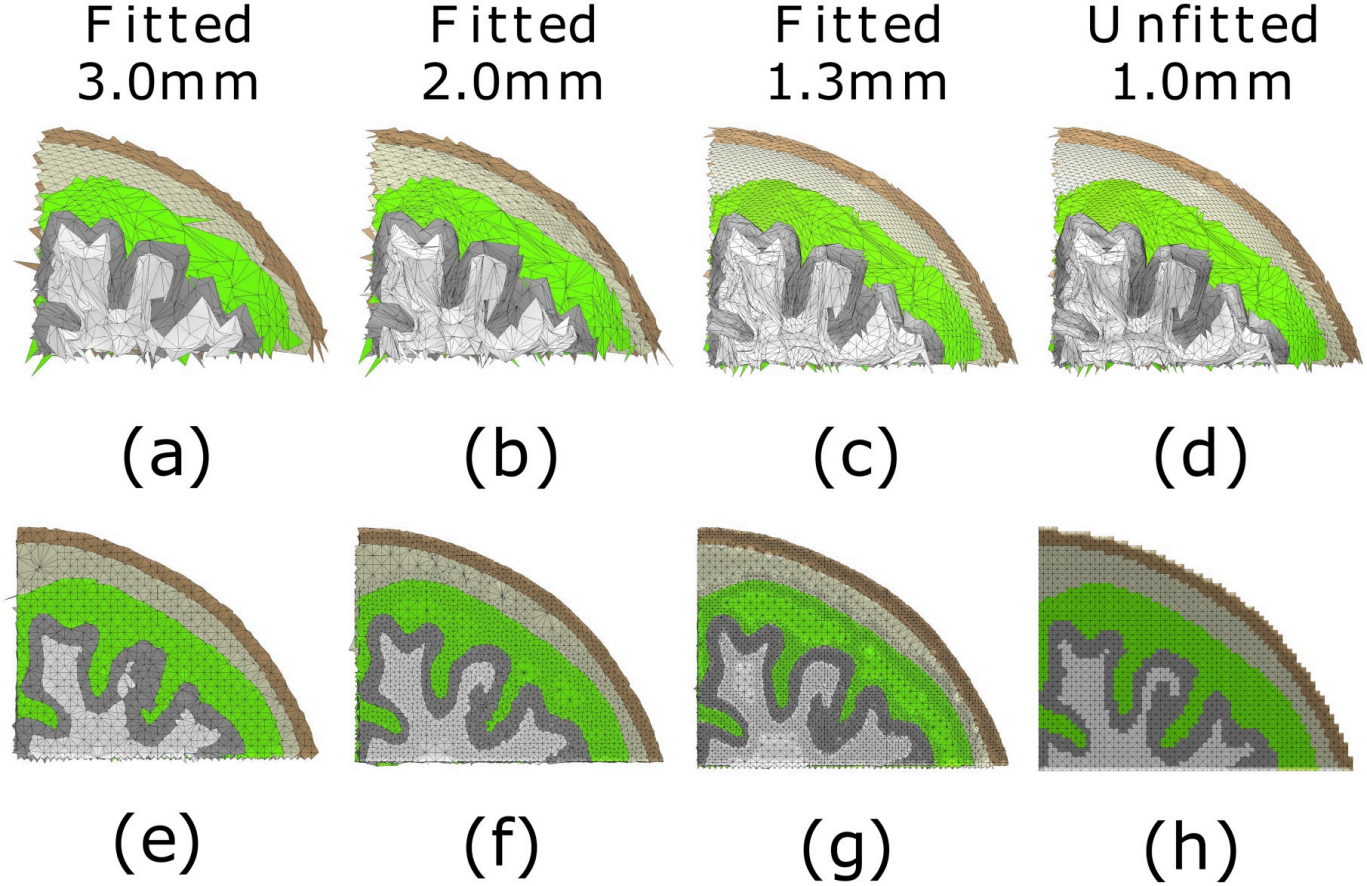
Mesh visualization: MRI-based model. Quadrants of the downsampled surface grids (a-c) and the boundary-fitted tetrahedral mesh (e-g) for 3.0, 2.0 and 1.3 mm (millimeter) mesh sizes, respectively. Surface grids (d) and unfitted tetrahedral mesh (h) for 1.0 mm mesh size are included for comparison. The presented layers are (top-bot): scalp (brown), skull (white), and grey matter. The presented layers (top-bot) are the scalp (dark brown), skull (light brown), cerebrospinal fluid (green), grey matter, and white matter.

The accuracy of the fitting is illustrated in Figs 7 and 8 for the spherical and MRI-based cases, respectively. The effect of surface refinement is visible in the case of a boundary-fitted 1.3 mm mesh; the resolution of the mesh decreases towards the interior in the compartments that share the refined boundaries, while in the case of regular mesh, it is the same everywhere.

**Fig 9:**
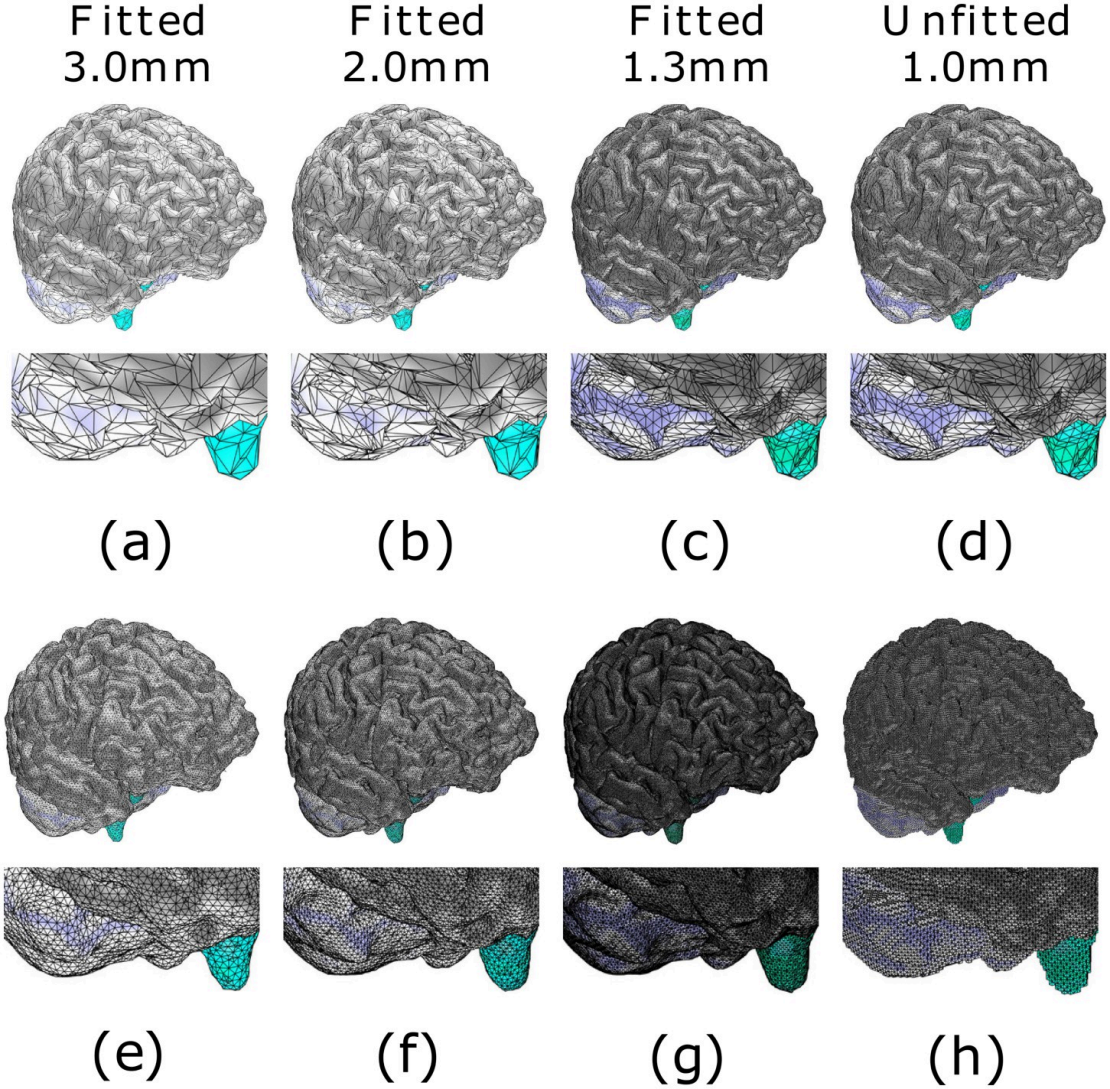
Superficial brain structures. Downsampled surface grids (a, b, c, d), Boundary-fitted meshes with 3.0, 2.0, and 1.3 mm (millimeter) mesh sizes (e, f, g) and unfitted mesh with 1.0 mesh size (h), respectively, for cerebrum (grey), cerebellum (light blue).

**Fig 10:**
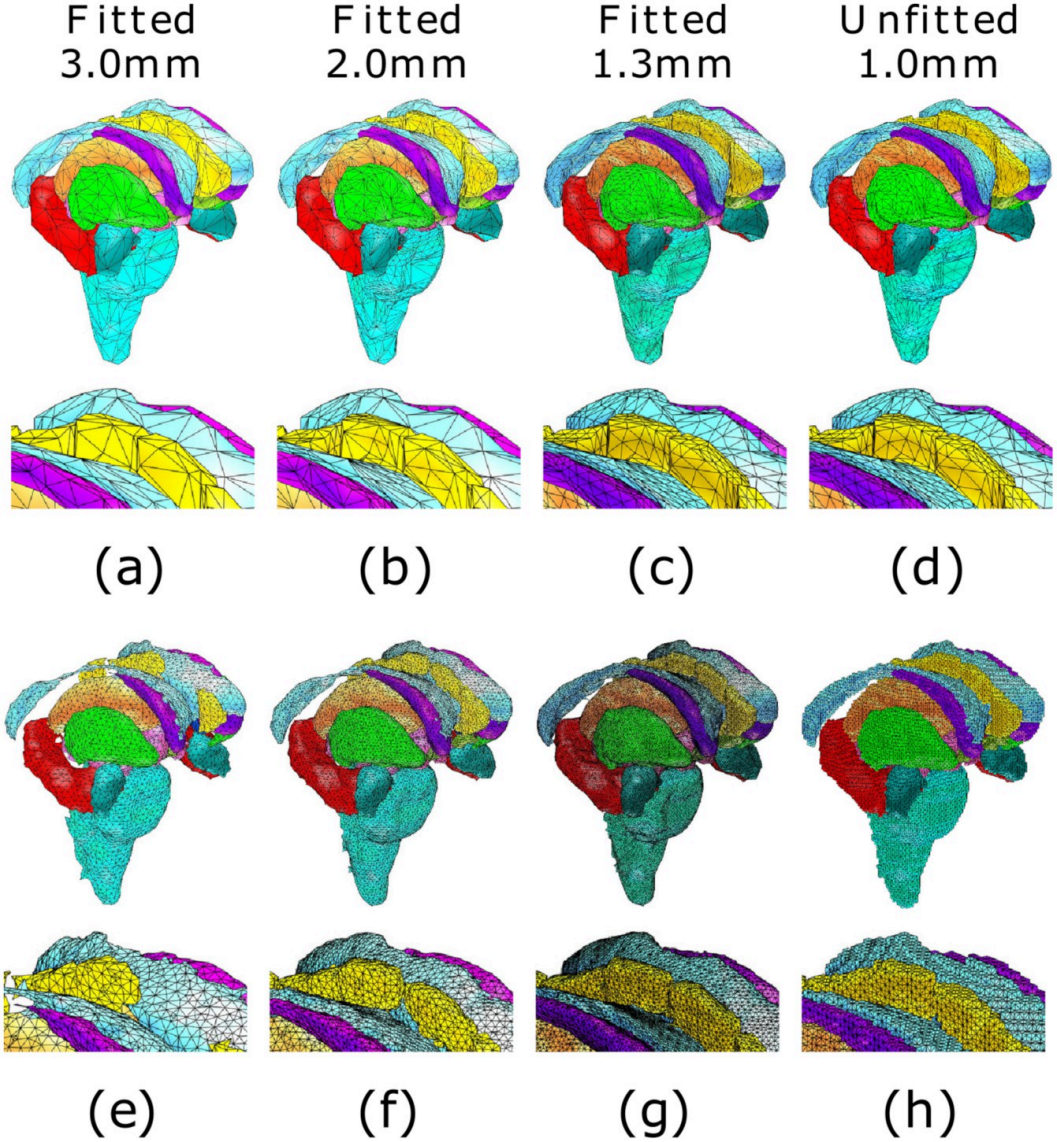
Subcortical structures. Downsampled surface grids (a, b, c, d), Boundary-fitted meshes with 3.0, 2.0, and 1.3 mm (millimeter) mesh sizes (e, f, g) and unfitted mesh with 1.0 mesh size (h), respectively, for brain stem (cyan), hippocampus (red), amygdala (dark green), putamen (light green), thalamus (orange), caudate (purple), ventricles (blue), and cingulate cortex (yellow). The focused image beneath illustrates that the coarse boundary-fitted 3.0 mm meshes and the unfitted mesh yield somewhat inaccurate results for the anatomical details, e.g., an incomplete cingulate cortex, compared to the finer 1.3 and 2.0 mm boundary-fitted meshes.

In Fig 9, we show FE meshing-based reconstructions of the cerebrum, cerebellum, and brain stem. Fig 10 displays the subcortical components, including the hippocampus, amygdala, putamen, thalamus caudate, ventricles, and cingulate cortex. An increase in the mesh resolution improves the shape and structure of the modeled tissues, which is particularly prominent for the caudal anterior area of the cingulate cortex, not fully reconstructed in the case of the 3.0 mm resolution, which we attribute to a comparably coarse initial resolution.

### EEG forward modeling

**Fig 11:**
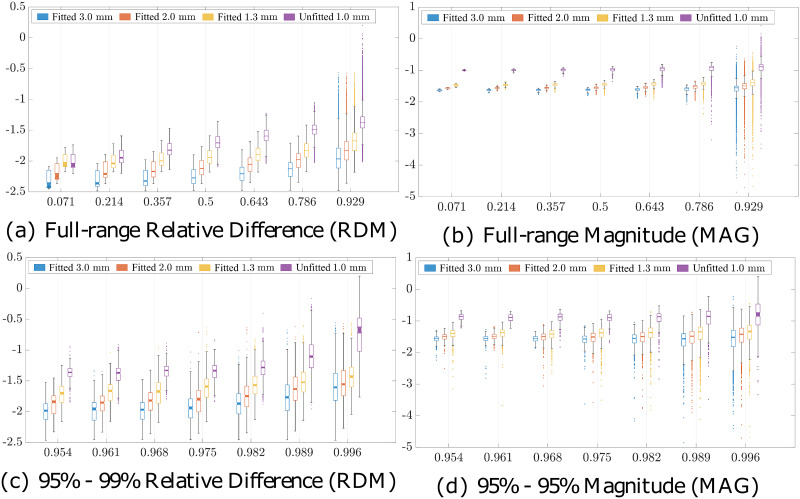
Lead field matrix accuracy. Accuracy of the lead field matrix evaluated at different eccentricities in Ary sphere, i.e. relative distances from the origin in the brain compartment using relative difference measure (RDM) and magnitude measure (MAG). The horizontal axis corresponds to the eccentricity (relative distance from origin with respect to the grey matter surface) and the vertical one to the difference measure (%) in question.

The RDM and MAG obtained with the spherical geometry are visualized in Fig 11. The boundary-fitted meshing enhanced the accuracy of lead field matrices as compared to unfitted meshing; the smallest differences were obtained in the boundary-fitted 1.3 mm case. For each lead field matrix, RDM had a median below 5%, while MAG was below 20% up to the eccentricity of 0.95. For the boundary-fitted meshes, these medians did not exceed 3 and 5%. With the finest boundary-fitted mesh, the upper limits of 3 and 4% were maintained for eccentricities up to 0.998. The effect of adapting was pronounced towards the high eccentricities, as is shown by the growing spread of the RDM and MAG distributions.

### Source localization

**Fig 12:**
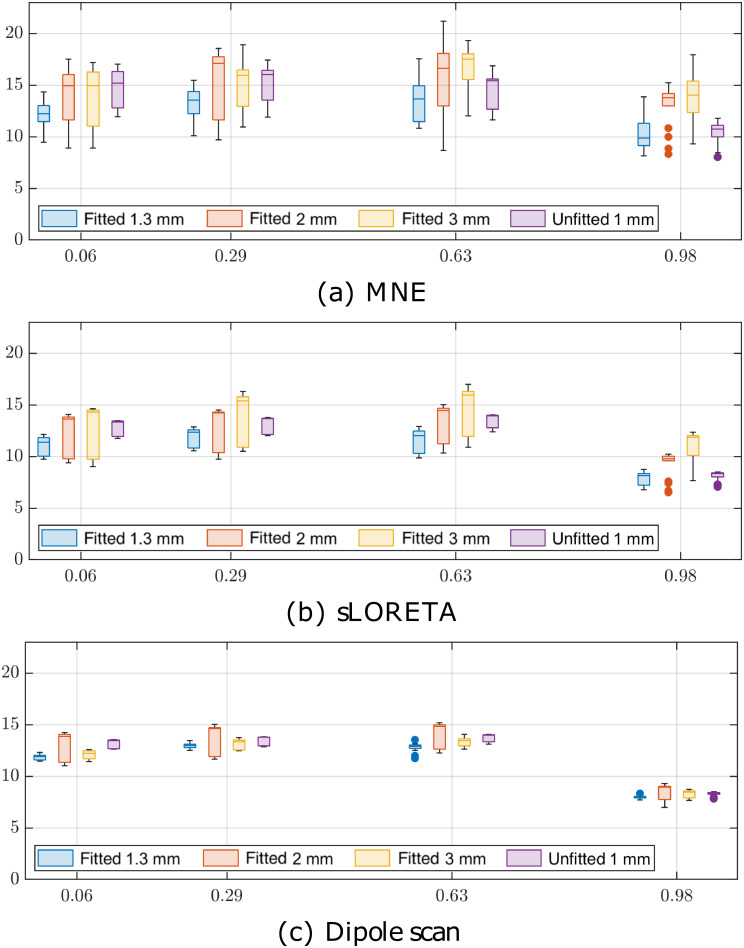
Source localization accuracy. The earth mover’s distance (EMD) measuring the amount of work required to transfer a distributional reconstruction into a dipole distribution (Dirac’s delta) using (a) minimum norm estimate (MNE), (b) standardized low-resolution brain electromagnetic tomography (sLORETA), and (c) dipole scan.

The source localization estimates for the spherical geometry and four different eccentricities, 0.06, 0.29, 0.63, and 0.98%, are shown in Fig 12. The relative mutual differences between the boundary-fitted and unfitted meshing approaches, measured via EMD, were observed to be less prominent in source localization as compared to forward modeling. These EMD differences also depend on the applied inverse method. Matching with the forward simulation results, the boundary-fitted 1.3 mm mesh provided the smallest median in each case, with a maximum of 3.0 mm marginal to the other cases. For MNE and sLORETA, the initial FE mesh resolution (3.0, 2.0, and 1.3 mm) seems to be the governing factor determining the differences in source localization accuracy, while for the dipole scan, such a tendency was not observed with the mutual differences between the methods being minor. In each case, the EMDs grow towards the center of the domain (median 8–14 mm in the vicinity of the boundary and 12–15 mm near the center), which is a natural consequence of the ill-posed nature of the source localization problem.

### SEP components P14/N22 and P22/N22

**Fig 13:**
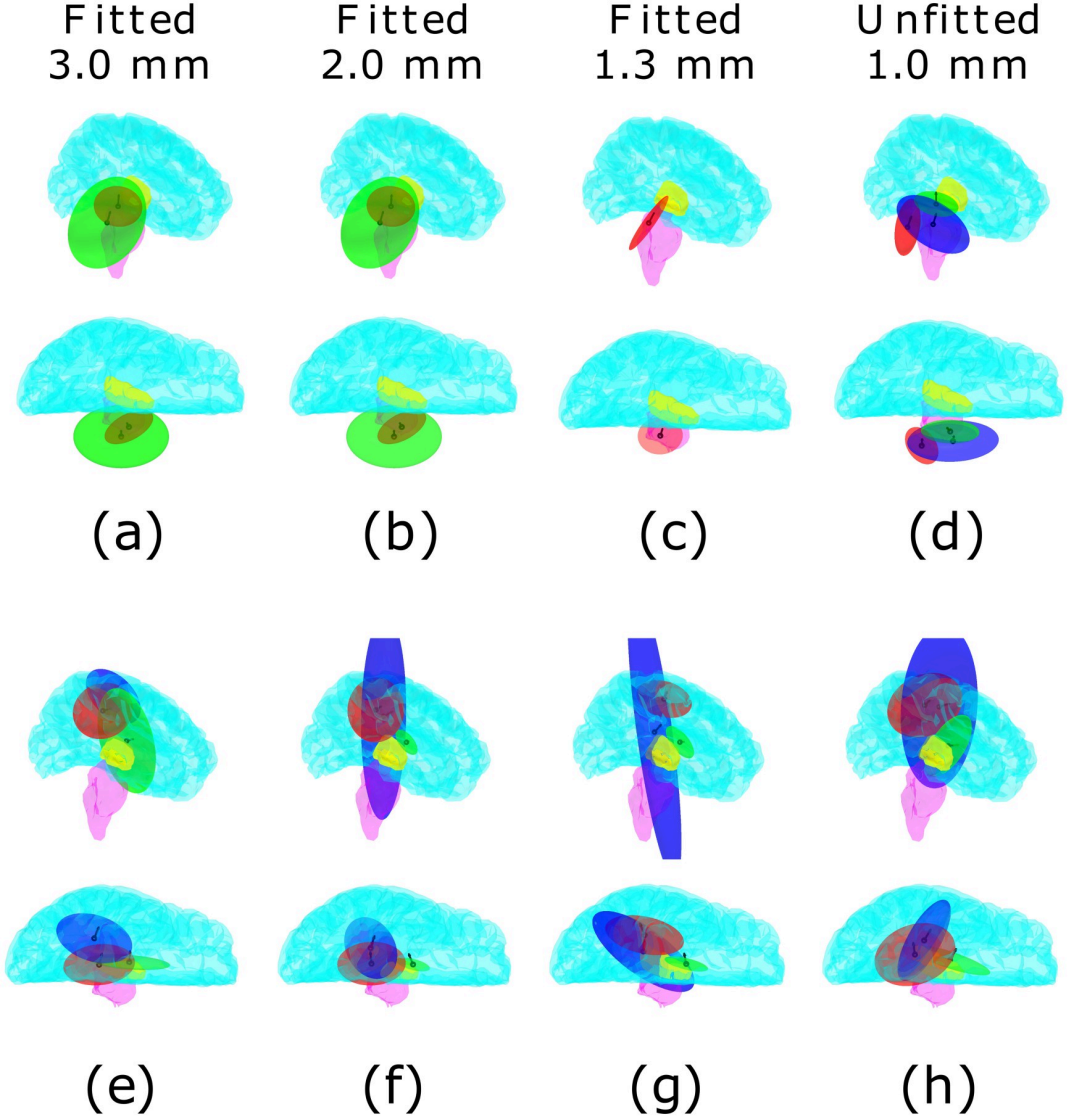
Clustering of simultaneous cortical and subcortical activity. Clusters obtained via Gaussian mixture modeling (GMM) for the P14/N14 (a-d) and P22/N22 (e-h) components of the experimental SEP dataset [17, 20]. Each cloud shows the ellipsoidal 90% credibility set of the corresponding cluster. Clouds are color-labeled according to their measured intensity levels in cubic/millimeters (mm^3^).

The GMM clusters corresponding to the SEP components P14/N22 and P22/N22 are shown in Fig 13. Table 4 describes the cluster volumes and their best-fitting cluster counts. Clusters have been color-labeled according to their intensities (R = red, G = green, B = blue) in descending order.

**Table 4:** Volume-value of the up-to-three obtained GMM-based clusters (R = Red, G = Green, B = Blue) ordered in descending order with respect to their intensity, measured in cubic millimeters (mm^3^).

	P14/N14 component			P22/N22 component		
Mesh size	R	G	B	cR	cG	cB
Fitted 3.0	889	34104	-	2612	4581	5697
Fitted 2.0	889	34104	-	2606	10	22317
Fitted 1.3	455	-	-	2353	23	46303
Unfitted 1.0	393	333	6537	9915	824	31988

The volume of the P14/N14 (R, G, B) and P22/N22 clusters (cR, cG, cB).

For P14/N14, a single cluster concentrated at the brain stem was found in the case of the boundary-fitted 1.3 mm mesh, which is in accordance with the physiological knowledge of the originator (see Somatosensory evoked potential data). Two clusters were found with boundary-fitted 2.0 and 3.0 mm meshes, while the unfitted one resulted in three reconstructed clouds, which is partially contrary to the knowledge of a single originator. The weaker clouds were larger in size, suggesting that they are due to modeling errors, as the actual originator is known to be well-localized in the brain stem.

For P22/N22, three clouds were obtained with each FE mesh. The distinction of the two most intense clouds, R and G, into cortical and thalamic components, was the clearest with the boundary-fitted 1.3 mm mesh. In the other meshes, the clusters were closer to each other or overlapped; in particular, the centroid of the most intense cluster was located deeper in the brain, suggesting that the reconstructed cortical and sub-cortical activity were partially mixed in the distribution found by sLORETA. The weaker clusters were larger in size, suggesting that they corresponded to modeling errors.

## Discussion

This study has demonstrated the open-source MATLAB-based Zeffiro Interface [13] capabilities to create a FE mesh [6] for EEG source localization using a multi-compartment model of the human head featuring cortical and subcortical compartments. The techniques applied in this study are based on nested compartment structures allowing a robust mesh generation for principally an arbitrary set of segmentated tissue boundaries. One advantage is the capability to generate a mesh regardless of intersecting segmentation boundaries, inhibiting the issues that can follow from intersecting surfaces when using a standard heuristic FE mesh generator [8–10].

To accurately model and locate the source of EEG signals, it is noteworthy to refine the FE mesh close to tissue boundaries. However, this process can be computationally demanding and can lead to memory limitations. In this study, we used a GPU-accelerated recursive labeling approach to reduce computational costs and create precise FE simulations with accurate segmentations and compartments.

We tested three different initial mesh resolutions (3.0, 2.0, and 1.3 mm) and compared them to a 1.0 mm unfitted mesh obtained by subdividing a regular hexahedral lattice into tetrahedra without additional refinement, smoothing, inflation, or optimization steps. With each resolution, the grey matter boundary was approximated with a sub-one-millimeter mean distance error. The unfitted regular mesh was observed to have the greatest spread with part of the error distribution extending above 1.0 mm, while for 2.0 and 1.3 mm boundary-fitted meshes the distribution was limited to values below. A boundary-fit superior to 1.0 mm was found to be a crucial criterion for reconstructing the anatomical details of the head segmentation. In particular, small subcortical details, such as the structure of the cingulate cortex, required the finest resolution to be labeled correctly. We consider the 2.0 mm mesh resolution, nevertheless, attractive from the practical point of view, as it seems to provide an appropriate overall trade-off between meshing accuracy and processing time, which increases along with the resolution.

The finest resolution outperformed the unfitted 1.0 mm mesh in EEG forward simulation and source localization applications. The median forward accuracy obtained with the finest mesh at 98% eccentricity was 1.1% RDM and 2.8% MAG. For comparison, the earlier studies utilizing the divergence conforming source model [4, 38] obtained approximately 0.3% RDM and 0.3% MAG with an optimized spherical Stok model [52] using a mesh created with Gmsh [10].

According to [53], the most precise estimates for source localization accuracy in spherical geometry are 9.2 and 12.8 mm for superior and deeper locations, respectively, with standard deviations of 4.4 and 6.2 mm. These estimates align with the median EMD (calculated using the finest boundary-fitted mesh) of 8–10 mm and 12–13 mm for superior and deeper locations, respectively, with a spread of 2.0 mm in each case in source localization. As compared to the results obtained with the finest boundary-fitted mesh, the source localization estimates obtained with unfitted 1.0 mm mesh lacked some accuracy with MNE and sLORETA, the deterioration being of maximally 4.0 mm considering median values. This deficiency falls in the standard deviation, suggested for the experimental case [53], and can be interpreted as a lattice or interpolation effect [4, 54], where sources that are more distant from each other than in a denser mesh are used in interpolating a given source. Namely, the differences observed are close to the initial mesh resolution in size.

The GMM clusters, including count, position, and size, obtained using the experimental SEP data [20], suggest that the resolution of the mesh can have a significant effect on the inverse estimates obtained with an MRI-based setting considering all the mesh resolutions examined in this study. Reflecting the physiological knowledge of the number, size, and positioning of the GMM clusters found [25, 44–51], a mesh refinements and boundary-fitting seem beneficial with respect to the reconstructions of P14/N14 and P22/N22 components. This observation affected especially the boundary-fitted 1.3 mm case and is in line with the other results of this study. Because of the sensitivity to modeling errors, the reconstructed subcortical activity of the P14 component was highly subject to the applied FE mesh. The sensitivity of P22/N22 not only follows from its depth contribution but also from the simultaneous cortical originator in the pre-, or post-central gyrus.

The GMM deviations induced by the FE mesh differences can be considered as an expected outcome, since the non-invasive distinguishability of subcortical activity is, in general, a relatively recent finding [15] and since non-invasively reconstructed distributions of P14/N22 and P22/N22 originators have been analyzed only recently in [17]. This observation highlights the importance of accurate forward modeling, suggesting that even a few percent differences in RDM or MAG can lead to significant distributional effects when reconstructing weakly distinguishable activity.

### Summary and outlook

Our present approach constitutes an independent, time-effective tetrahedral mesh generator, free of supplementary pre-processing aspects. Based on our results, we are confident that a relatively fine sub-one-millimeter resolution might be necessary to completely distinguish the deeper structures as they appear in FreeSurfer’s Aseg atlas, justifying the examination of denser meshes and GPU acceleration as a way to speed up the labeling process. Moreover, the results that a sub-one-millimeter multi-compartment mesh can be necessary in some EEG applications, for example, in the non-invasive localization of early SEP originators.

While advanced toolboxes built upon well-known mesh generators have been recently applied for the human head, possibly most prominently iso2mesh [11, 55], to the best of our knowledge, there is currently no other EEG source localization pipeline that generates a boundary-fitted finite element mesh for a head segmentation including a complete set of subcortical structures. Highlighting the achievement of this study in modeling those, a recent effort [18] on non-invasive detection of subcortical brain activity was limited to a maximally six-compartment FE model. Thus, in contrast to the present results the subcortical structures, likewise segmented via FreeSurfer, were not fully distinguished as separate compartments in the FE mesh of [18], which was created using iso2mesh and the Computational Geometry Algorithms Library (CGAL). In general, a mesh with absent subcortical structures can be considered as the current standard in brain source imaging and stimulation, for instance, in iso2mesh- [11, 56] and SimBio-Vgrid-driven studies [57, 58].

Our future work will include further tests with experimental data and methodological and computational considerations, e.g., possibilities to speed up the surface extraction process, which is a major contributor to the total meshing time and involves complex indexing operations. The present meshing approach will be applied in the further development of advanced FEM forward simulation tools such as Duneuro [14] or packages for analyzing brain activity, e.g., the current implementation platform ZI [13] or the well-known Brainstorm [59] whose forward model has been built initially upon the boundary element method. State-of-the-art FE mesh adaptation techniques [60, 61] based on advanced error estimators constitute an important future work direction to further enhance the accuracy of meshing and the related forward model.

## Supporting information

S1 Appendix (ZIP)
