# Supplementary material for: Multi-compartment head modeling in EEG: Unstructured boundary-fitted tetra meshing with subcortical structures
Source: PLoS One. 2023 Sep 20;18(9):e0290715. doi: 10.1371/journal.pone.0290715 (PMC10511141; doi:10.1371/journal.pone.0290715)
Supplement: S1 Appendix — (ZIP) [file pone.0290715.s001.zip › S1_Appendix.pdf]

Supporting information

S1 Appendix. Meshing Algorithm. This section describes the pseudocode of the tetra meshing algorithm applied in this study. The actual algorithm is available as a part of the open-source MATLAB-based Zeffiro Interface (ZI) toolbox (https://github.com/sampsapursiainen/zeffiro_interface), in particular, release 5.17 corresponds to this study. The script matching the pseudocode below has been included in the file script/zef_meshing_example.m. To run the code, first add the scripts folder to MATLAB's path.

```
procedure BOUNDARY-FITTED MESH
    P.mesh_resolution = <VALUE >
    P.mesh_smoothing = TRUE
    P.refinement = TRUE
    P.refinement_surface = TRUE
    P.refinement_surface_compartments = <VALUE >
    return P                                                    ▷ (struct)

    if downsample_surfaces == TRUE then
        procedure ZEF_DOWNLOAD_SURFACES
            input: P, k                          ▷ (k is the number of compartments)
            while k ≠ number_of_compartments do
                Perform downsample surface (triangulation and tetras)
                k = k++
            end while
            output: P
        end procedure
    end if

    procedure HEXA MESHING
        input: P
        Perform: hexahedral → tetrahedral subdivisions
        Perform: labeling
        return: unfitted mesh
    end procedure

    procedure RE-LABELING
        input: unfitted mesh, P, g          ▷ g = [0] CPU or [1] GPU parallelization
        if g == 1 then
            P.use_GPU == TRUE
        else
            P.use_GPU == FALSE
        end if
        if  enable fit == FALSE then
            Perform: solid angle labeling
            return: refined mesh
        else
            while Convergence achieved == FALSE do
                Perform: solid angle labeling
                Perform: surface extraction
                Perform: re-labeling
            end while

            Input: refined mesh
            Perform: Taubin smoothing method
            Perform: Inflation
            Perform: Delaunay turns
            return: fitted mesh
        end if
    end procedure

end procedure
```
